# Polymerization Stress Development in Dental Composites: Effect of Cavity Design Factor

**DOI:** 10.3390/ma2010169

**Published:** 2009-03-13

**Authors:** Joseph M. Antonucci, Anthony A. Giuseppetti, Justin N.R. O’Donnell, Gary E. Schumacher, Drago Skrtic

**Affiliations:** 1 Polymers Division, National Institute of Standards and Technology, Gaithersburg, 20899, MD, USA; E-Mail: joe.antonucci@nist.gov; 2 Paffenbarger Research Center, American Dental Association Foundation, Gaithersburg, 20899, MD, USA; E-Mails: tony.giuseppetti@nist.gov (A.G.); justin.odonnell@nist.gov (J.D.); gary.schumacher@nist.gov (G.S.)

**Keywords:** Amorphous calcium phosphate, cavity design factor, composite, resin, polymerization stress, tensometry

## Abstract

The objective of the study was to assess the effect of the cavity design factor (C-factor) on polymerization stress development (PSD) in resin composites. An experimental resin (BT resin) was prepared, which contained 2,2-bis[*p*-(2’-hydroxy-3’-methacryloxypropoxy)phenylene]propane (B) and triethylene glycol dimethacrylate (T) in 1:1 mass ratio, and an activator for visible light polymerization. An experimental composite with demonstrated remineralizing potential was also formulated by inclusion into the BT resin of zirconia-hybridized amorphous calcium phosphate (ACP) filler at a mass fraction of 40 % (BT/ACP composite). A commercial glass-filled composite (TPH) was used as a control. To assess the effect of the test geometry on PSD, C-factor was systematically varied between 0.8 and 6.0 by varying the height of the cylindrical composite specimens. The measured PSD values obtained by cantilever beam tensometry for specimens with variable C-factors were normalized for mass to specimens with a C-factor of 1.33 (h=2.25 mm) as controls to give calculated PSD values. Degrees of vinyl conversions (DC) attained in the TPH control and in the experimental BT/ACP composites were measured by near-infrared spectroscopy. In both the TPH and BT/ACP composite series, PSD_calc_ increased with the increasing C-factor, confirming the hypothesis that the C-factor value influences PSD values. The higher PSD_meas_ and PSD_calc_ values for the experimental BT/ACP composite compared to the commercial TPH composite probably reflect differences in the type and mass of the resin and filler phases in the two types of composite. These differences also account for the observed variation (21 %) in DC attained in a BT/ACP composite 2 h after cure (69.5 %) and in the DC of the TPH composite (57.5 %) having the same C-factor. The cavity design factor seems to play a key role in influencing the PSD of bonded composites, but other factors such as composite mass and composition also must be considered for their effects on PSD.

## 1. Introduction

Since the introduction of dental composites into dentistry in the 1960s [1], considerable developments in filler technology, resin and polymerization initiation systems, as well as improvements in the adhesion of dental composites to tooth structures have significantly improved their properties and expanded their clinical utility. However, in spite of improvements in bonding properties, micro-leakage and gap formation, primarily at the dentin/composite interface, remain major weaknesses of these materials [2]. The polymerization of methacrylate-based dental composites is usually accompanied by significant shrinkage and the production of internal stresses that depend on both material and processing variables [3,4]. Despite many investigations, the highly complex phenomenon of polymerization shrinkage development in polymeric dental restorative materials is not yet fully understood and remains a significant clinical concern [5]. This phenomenon becomes even more complex when the composite is bonded into cavities of variable configurations. During the photo-polymerization of restorative composites, a complex network involving the resin and silanized filler phases quickly forms at the gel point. The composite’s elastic limit reaches a level that does not allow enough relaxation to occur to compensate for the reduction in volume, and rapid buildup of stress occurs both in the composite and at the composite/tooth interface. Any additional polymerization shrinkage beyond the gel point adds to this internal stress that develops in the polymer matrix and its interfaces with the filler particles. For composites bonded to enamel/dentin, polymerization shrinkage is constrained and polymerization stress development (PSD) becomes more complex due to the generation of interfacial stresses involving tooth structures, usually unevenly distributed along the cavity walls and the bonded composite surface [6,7].

A number of material as well as processing factors can contribute to PSD in composites. Filler type and content, resin type and composition, and mode of polymerization determine the amount of volumetric shrinkage, elastic modulus and PSD of the composite [8]. The polymerization process is affected by the type and concentration of initiators, e.g. chemical vs. photochemical, which determine reaction kinetics and degree of vinyl conversion (DC, [9]). PSD values also vary according to the ratio of the bonded to the unbonded (free) surface area of the composite in a cavity, i.e., the configuration or C-factor [10,11,12]. Although the PSD in photopolymerized dimethacrylate monomer systems has been studied quite extensively [5,6,8,9,13,14,15,16], a fuller understanding of the kinetics of polymerization shrinkage and the accompanying stress is still lacking. Similarly, how cavity configuration affects the performance of bonded composite restoratives, adhesives and sealants [10,11,12,17,18,19] also needs to be better understood. Feilzer *et al*. [19] have hypothesized that a larger free surface area (lower C-factor value) in the restorative composite would lead to lower PSD values by allowing greater plastic deformation to occur during polymerization before the gel point is reached.

The aim of this study was to test the above hypothesis by assessing by tensometry the effect of the cavity design factor (C-factor) on PSD in resin composites bonded to a silanized glass substrate as a model for composites bonded to surfaces of a dental cavity. Specifically, the objectives were to study how C-factor variations affect the PSD in a typical amorphous calcium phosphate (ACP)-based composite with demonstrated remineralizing potential [20,21,22] and a typical commercial glass-filled composite using a cantilever beam based tensometer [23].

## 2. Results and Discussion

**Figure 1:**
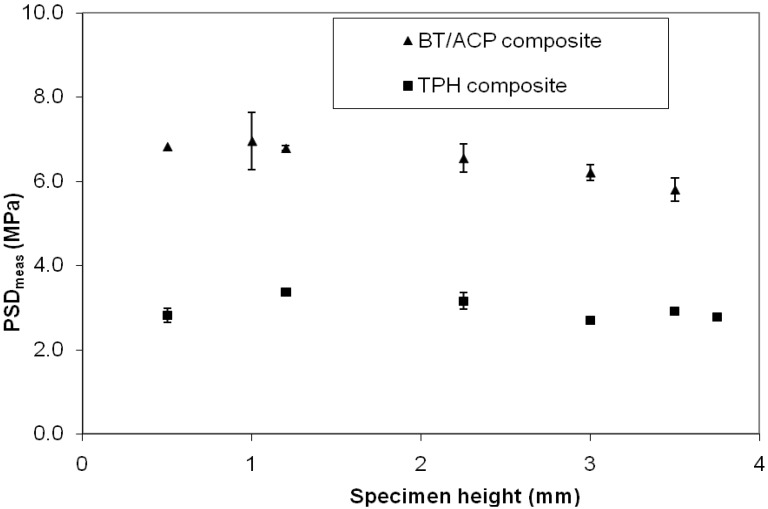
PSD_meas_ as a function of specimen height. Indicated are the mean values ± one standard deviation (SD). Number of runs n= 3/experimental group.

PSD_meas_ and PSD_calc_ data obtained for the BT/ACP and TPH composite specimens for a range of cavity configuration factors (C-factors) between 0.8 and 6.0 (composite height from 3.75 mm to 0.50 mm) are given in Table 1. The PSD_meas_ varied between 5.80 MPa and 6.96 MPa in the BT/ACP composite series and between 2.78 MPa and 3.37 MPa in the TPH composites. For any given C-factor, the value of PSD_meas_ in BT/ACP composites more than doubled the value obtained in the corresponding TPH control. Also, data scattering was significantly lower for the TPH compared to BT/ACP composite specimens (SD values ranging from 0.05 MPa to 0.19 MPa vs. 0.06 MPa to 0.68 MPa). Plotting PSD_meas_ values obtained either for BT/ACP or for the TPH composite specimens as a function of composite specimen height showed practically no correlation between the two (Figure 1).

**Table 1:** Measured PSD (mean value ± SD of three repetitive measurements) and the corresponding calculated PSD for BT/ACP and TPH composites as a function of C-factor.

C-Factor	Composite Height (mm)	BT/ACP Composite **PSD** (MPa) Measured Calculated	TPH Composite **PSD** (MPa) Measured Calculated
0.80	3.75	nd	2.78 ± 0.07 1.67
0.86	3.50	5.80 ± 0.55 4.39	2.91 ± 0.05 1.87
1.00	3.00	6.21 ± 0.27 4.66	2.70 ± 0.07 2.03
1.33	2.25	6.55 ± 0.19 6.55	3.16 ± 0.19 3.16
2.50	1.20	6.79 ± 0.34 12.73	3.37 ± 0.08 6.32
3.00	1.00	6.96 ± 0.06 14.73	nd
6.00	0.50	6.83 ± 0.68 26.09	2.82 ± 0.17 12.69

**Figure 2:**
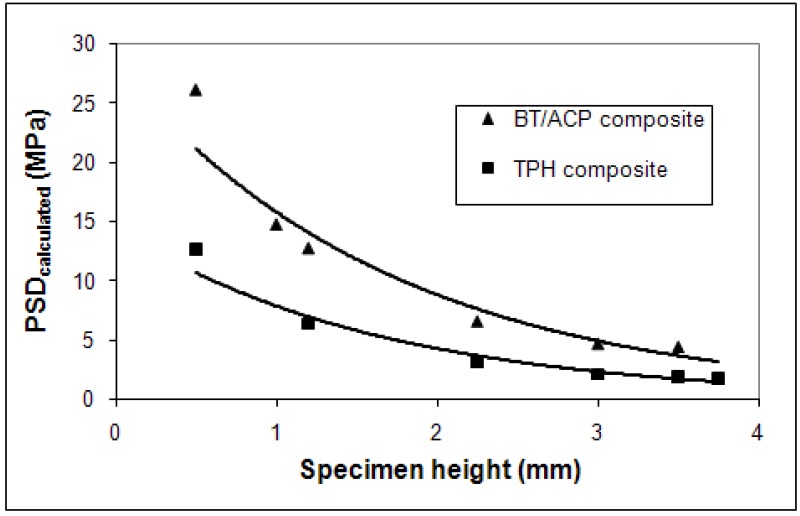
Functional dependence of PSD_calc_ (mean value ± SD) on the specimen height. The average PSD_calc_ values calculated from the mean PSD_meas_ data (n = 3/group).

However, the corresponding PSD_calc_ values decreased with the increasing specimen thickness (height, h) for both the experimental and control groups (Figure 2) according to the following exponential functions (R^2^ is the correlation coefficient):

PSD(ACP)_calc_ = 28.3^.^e^-0.58h^ (R^2^ =0.9542) (1)

PSD(TPH)_calc_ = 14.5^.^e^-0.61h^ (R^2^ =0.9680) (2)

**Figure 3:**
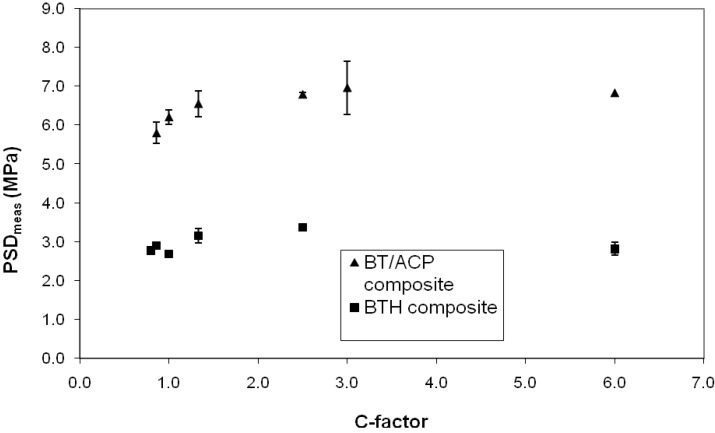
PSD_meas_ (mean value ± SD) as a function of specimen height.

**Figure 4:**
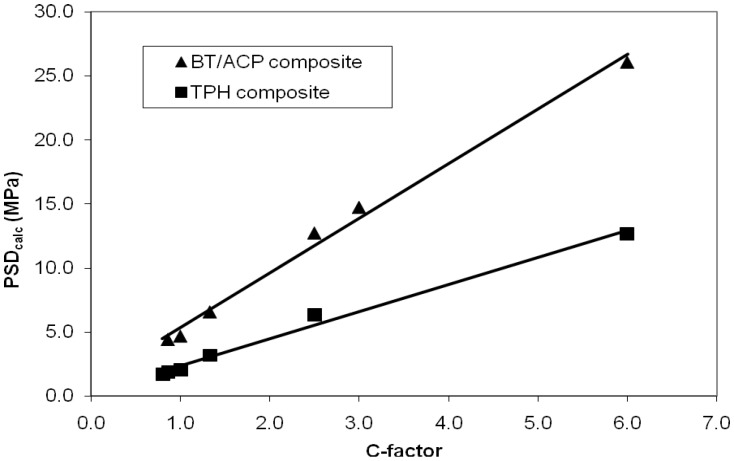
Functional dependence of PSD_calc_ on the cavity configuration factor. The average PSD_calc_ values calculated from the mean PSD_meas_ data (n = 3/group).

Similarly, no correlation existed between the PSD_meas_ and C-factor for both types of composites (Figure 3). On the other hand, PSD_calc_ and the C-factor for both the BT/ACP and TPH composites (Figure 4) showed linear correlations that can be described by the following equations, respectively:

PSD(ACP)_calc_ = 4.28 ^.^ C-factor + 1.05 (R^2^ = 0.9923) (3)

PSD(TPH)_calc_ = 2.13 ^.^ C-factor + 0.20 (R^2^ = 0.9903) (4)

**Figure 5:**
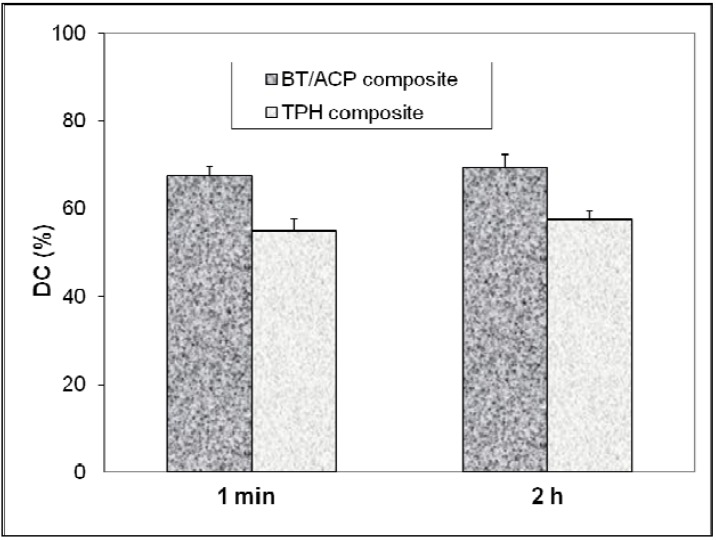
Degree of conversion (DC) attained 1 min and 2 h after visible light-curing of the BT/ACP and TPH composites, with the mean values of three repetitive measurements in each group; SDs are indicated.

DC values attained in the BT/ACP and TPH composites at 1 min and 2 h after light-cure, shown in Figure 5, clearly indicate higher (21 % to 23 %) vinyl conversion in the BT/ACP composites compared to TPH control.

In this study an attempt was made to mimic the constrained polymerization shrinkage and stress development that occurs in composites bonded to tooth structure. The silanized quartz surfaces mimic bonding to flat dental surfaces via the use of dental adhesives also designed to chemically bond to composite restoratives or inlays. Studies of PSD as a function of specimen thickness in applications simulating the cementation of inlays, i.e. very thin layer of adhesive or composite [24,25], have shown a substantial disparity in stress with specimen height. For the range of specimen thickness examined in our study (0.5 mm to 3.75 mm) no correlation was found between the PSD_meas_ and the specimen thickness for both types of composites examined. One may attribute such findings to the lack of instrument sensitivity to detect differences in PSD for composite specimens over the range of C-factors studied. Watts and Satterthwaite [26] have, however, indicated that at specimen thicknesses more akin to the resin-composite direct restorations, i.e., at thicknesses between 0.8 mm and 1.5 mm, the variations in PSD tend to be minimal and are less affected by the instrument compliance. They have shown that the uniaxial shrinkage stress/mass increases linearly with the increasing C-factor (decreasing height of a specimen). Similar findings were reported by Lee et al. [27]. In this study (Table 1; Figure 4) PSD_calc_ was directly proportional to C-factor confirming the original hypothesis. However, PSD_meas_ data showed no correlation with either specimen thickness (height, h; Figure 1) or C-factor (Figure 3). The apparent discrepancy between the two data sets (PSD_meas_ vs. PSD_calc_) remains unexplained. Eq. (2) implies a simple relation between C-factor and ‘h’ that should be reflected in PSD_meas_.

Consistently higher PSD values for the experimental BT/ACP composite compared to the TPH control composite reported in this study are not unexpected; the higher DC attained in the ACP composite coupled with a significantly lower filler content would be expected to lead to higher shrinkage upon polymerization and more stress development compared to the less converted and more highly filled TPH composites. Not only the mass but also the type of filler used (a mass fraction of 40 % ACP without surface treatment in BT/ACP composites vs. a mass fraction of 78 % silanized glass filler in TPH composite) can affect PSD. Also the greater translucency of the ACP composite compared to TPH may enhance the degree of radiance of the former and contribute to the observed higher DC. Another critical factor is the composition of the resin phase (Bis-GMA/TEGDMA (BT/ACP composite) vs UDMA-modified Bis-GMA in TEGDMA (TPH composite)). Generally, the urethane-modified Bis-GMA oligomer would be expected to shrink less than Bis-GMA/TEGDMA. With respect to the PSD, it is generally beneficial to have high levels of fillers in composites since their contribution, even when treated with coupling agents (e.g. silanized), to polymerization shrinkage is minimal [28] but may contribute to PSD by increasing the elastic modulus. The filler content of the resin-based composites has indeed been indicated as a major determining factor for PSD [10]. On the other hand, the internal interfacial stress during the setting contraction of a resin composite is proportional to elastic modulus (Young’s modulus) of the material [29]. Other material factors being equal, the most rigid material, i.e., the one with highest modulus will show the highest PSD with the progression of polymerization reaction [30]. In view of the lack of correlation that existed between the PSD_meas_ and C-factor for the composites, it could be informative to develop means to plot and correlate the temperatures of the composites’ exothermic polymerization, and the cooling that follows, along with the measurement of the PSD [3]. 

## 3. Experimental Section

### 3.1. Resin formulation, ACP filler synthesis and characterization, composite preparation

The experimental resin was formulated from commercially available 2,2-bis[*p*-(2’-hydroxy-3’-methacryloxypropoxy)phenylene]propane (Bis-GMA) and triethylene glycol dimethacrylate (TEGDMA) monomers in a 1:1 mass ratio (designated BT resin). Bis-GMA and TEGDMA were used as received from the manufacturer (Esstech, Essington, PA, USA) without additional purification. BT resin was photo-activated by the inclusion of a mass fraction 0.2 % camphorquinone and a mass fraction 0.8 % ethyl 4-*N, N*-dimethylaminobenzoate. 

The ACP remineralizing composite (designated BT/ACP composite) was formulated with a zirconia-hybridized amorphous calcium phosphate (Zr-ACP) filler (median particle diameter d_m_ = (5.7 ± 2.2) μm). Composite paste was prepared by hand spatulation by combining mass fractions of 40 % Zr-ACP and 60 % BT resin. Zr-ACP was synthesized and characterized as detailed earlier [20]. The volume filler fraction of the experimental material is 30 %. Its shade is B1 compared to the VITAPAN Lumin Vacuum shade guide (Vita Zahnfabrik, Bad Säckingen, Germany). The observed slightly higher translucency is attributed to the relatively low filler content by volume. A commercial composite (TPH^3^ Micro Matrix Restorative: lot #070403, shade A1, Dentsply-Caulk, Milford, DE, USA) was used as control in this investigation. TPH^3^ consists of a visible-light activated, urethane-modified Bis-GMA, ethoxylated-Bis-GMA and TEGDMA (1 : 1 : 1 mass ratio). The composites contains mainly a barium boron aluminum silicate glass at a 78 % mass fraction level (filler volume fraction is approx. 57 %).

### 3.2. Polymerization stress development (PSD) measurements

**Figure 6:**
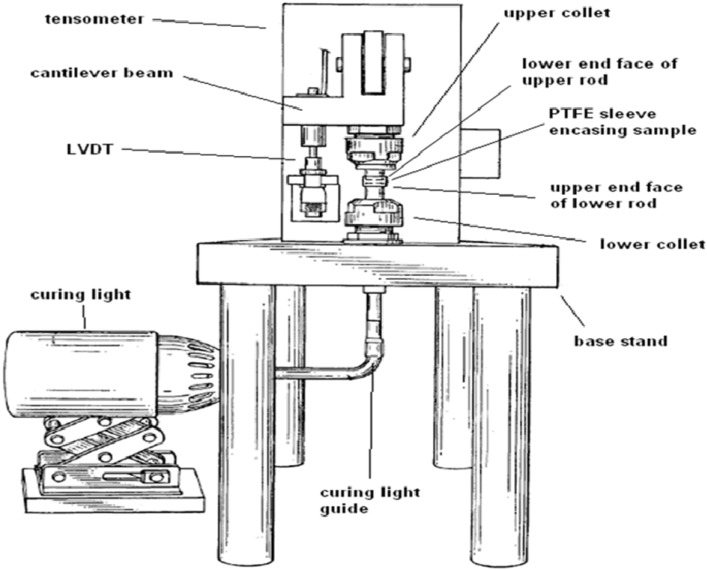
Schematic of a tensometer such as the one utilized in the study. In the present study, Tygon sleeves (instead of PTFE) encased the samples, and their inner surfaces represented the unbonded areas.

**Figure 7:**
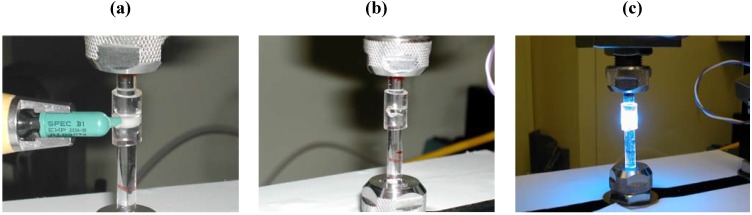
Positioning tygon (a), filling the mold (b) and light curing of the specimen (c) in the tensometer.

PSD was quantified by utilizing a computer-interfaced, cantilever beam tensometer developed at Paffenbarger Research Center, ADAF, at NIST, Gaithersburg, MD, USA (Figure 6, Figure 7). For a rectangular prismatic cantilever beam of a linearly elastic material with a small deflection, which is under a concentrated normal load F, the displacement at the end of the cantilever beam is defined by the following expression:

ε/F = 2a^2^(3L-a)/Ebd^3^ (5)

In Equation (1), ε is the displacement at the beam end (μm); E is the Young’s modulus of the cantilever beam (MPa); F is the load (N) needed to generate the displacement ε; L is the total beam length (cm); a is the distance from the load-applied position to the end of the beam (cm); b is the width of the beam (cm) and d is the height of the beam (cm). The deflection of the cantilever beam was measured with a linear variable differential transformer. The force was calculated from a beam length (12.5 cm) and a calibration constant (3.9 N/μm). PSD was obtained by dividing the measured force by the cross sectional area of the sample (diameter = 6 mm).

To systematically evaluate effects of different configurations on PSD, the heights (h) of unpolymerized composite cylindrical specimens were varied between 0.5 mm and 3.75 mm to give C-factors ranging from 6.0 to 0.8. For a circular quartz rod of diameter 2r and a specimen of height h, C-factor was calculated as the ratio of bonded composite area (the silanated ends of the silica rods) to the unbonded area (the compliant plastic enclosure) according to the expression:

C-factor = 2πr^2^/2πrh = r/h (6)

The composites were irradiated through the lower quartz rod with a visible light (Spectrum Curing Unit, Dentsply-Caulk, Milford, DE, PA, USA) for 60 s to initiate polymerization, and the PSD was then measured after 60 min. The light intensity, measured by a Demetron Model 100 radiometer (Demetron Research, Danbury, CT, USA) was (510 ± 25) mW/cm^2^ at the upper end of the top quartz rod where the sample was bonded.

A recent study utilizing a different cantilever beam tensometer [31] demonstrated the importance of also considering the mass of the cylindrical composite specimen as well as its C-factor. Because our specimens all had the same diameter, h becomes the determinant of the composite specimen mass as well as its C-factor. The measured PSD values (PSD_meas_) for specimens with variable heights were normalized for mass to a control specimen with h = 2.25 mm (C-factor = 1.33) to give calculated PSD values (PSD_calc_) using the following expression:

PSD_calc_ = PSD_meas_^.^ (h_control_/h_variable_) (7)

A minimum of three measurements were made for each experimental group.

### 3.3. Degree of vinyl conversion (DC)

The DC attained in the TPH control composite and in the experimental BT/ACP composites was measured at 23 °C by near-infrared (NIR) spectroscopy [32]. NIR scans (Nicolet Magna 550, Nicolet Inc., Madison, WI, USA) were taken before photo cure and 1 min and 2 h post-cure of composites with a thickness of approx. 3.0 mm (C-factor = 1.00 if h = 3.0 mm) and compared. DC corresponded to the bulk of composite and was defined as the % change in the integrated peak area of the 6165 cm^-1^ absorption band related to the first overtones of the =C-H stretching vibrations of the methacrylate vinyl group (=CH_2_) before and after photo-polymerization,. It was calculated utilizing the following formula:

DC = {1-[(area/thickness)_polymer_/(area/thickness)_monomer_]}x100 (8)

By measuring the thickness of monomer/polymer specimens the need to use an invariant absorption band as an internal standard was circumvented.

### 3.4. Statistical data analysis

One standard deviation (SD) is identified in this paper for comparative purposes as the estimated standard uncertainty of the measurements. These values should not be compared with data obtained in other laboratories under different conditions. Experimental data were analyzed by analysis of variance (ANOVA; α = 0.05). Significant differences between the groups were determined by all pair-wise multiple comparisons (Tukey-test).

## 4. Conclusions

The cavity design, i.e., configuration factor (C-factor) needs to be considered in minimizing the polymerization stress development and, in turn, improving the quality of the interface between the composite and tooth structures. However, material characteristics of the composite (filler type and content, resin type, initiator system) as well as those of the dental adhesive system also can influence the development of internal and interfacial stresses. Tensometry has the potential for aiding in optimizing the material and processing factors that can lead to the development of polymeric materials with favorable PSD values.
